# A deep learning approach to county-level soybean yield forecasting using large-scale environmental data

**DOI:** 10.3389/frai.2026.1914697

**Published:** 2026-09-17

**Authors:** Bennet Chen, Wei Zhang

**Affiliations:** 1 Little Rock Central High School, Little Rock, AR, United States; 2 Department of Mathematics and Statistics, University of Arkansas at Little Rock, Little Rock, AR, United States

**Keywords:** climate change, climate resilience, crop yield forecasting, deep neural network, interpretable machine learning, SHAP analysis, soybean yield

## Abstract

**Introduction:**

Agricultural systems continue to be disrupted by climate change, making crop yield forecasting a critical component towards informed agricultural planning. However, current yield forecasting approaches often lack in scale and are hard to interpret agronomically within real world scenarios. To fill the gap, this study developed an interpretable soybean yield forecasting framework using quantifiable county-level climate and soil attributes at scale for early, accurate yield predictions months ahead of harvest.

**Methods:**

Here, 388 climate, soil, and elevation attributes were collected from a ~100-year time span over the continental US. After preprocessing, Shapley Additive Explanation (SHAP) values were computed for each factor to aid in the interpretability of forecasts within the framework. Using the combined climate factors, a deep learning neural network was tuned and constructed to produce yield forecasts in May, June, July, and August.

**Results:**

On a hold-out test set, these forecasts produced robust predictions, indicating the framework can reliably predict yields up to five months ahead of harvest. This study also indicated the importance of several environmental attributes towards yield forecasts; specifically, extreme temperatures during later summer months (DX90, EMXT) were found to have outsized negative contributions towards soybean yields, while timing of heat was also identified as a potential driver of yield (DYXT).

**Discussion:**

Overall, these findings indicate that our interpretable DNN model is capable of providing reliable, in-season soybean yield forecasts as early as May, offering a robust tool for agricultural stakeholders to adjust management strategies well before harvest.

## Introduction

Due to the effects of anthropogenic climate change, it is estimated that agricultural productivity growth has slowed by around 21% since 1961 (Ortiz-Bobea et al., 2021). Yet, food demand continues to rise, with food demand projected to increase as much as 56% by 2050 (Van Dijk et al., 2021). With the global population recently reaching 8 billion and continuing to rise (Jain et al., 2023), accelerating agricultural productivity and enhancing climate resilience in agriculture in the context of climate change has become critical for sustainable development and for enabling worldwide food security (Borlaug, 2002).

Global climate change primarily impacts agriculture through the disruption of weather patterns, which serve as primary drivers of crop productivity. Weather shifts caused by climate change have been characterized by rising average temperatures, accelerated hydrological cycles, and greater volatility in precipitation events (Intergovernmental Panel on Climate Change (IPCC), 2023; Huntington, 2006; Pendergrass et al., 2017). These climate shifts over the past several decades have had immense impacts on agricultural productivity, both globally and within the US.

The impact of climate change has been especially imperative for soybeans (*Glycine max*), which have emerged as an important staple crop within the food supply system due to their high protein content, nutritional quality, and versatility as a plant-based food source (Messina, 2022). Yet, although yields have more than doubled over the past century (Masuda and Goldsmith, 2009), soybeans have been particularly sensitive to weather fluctuations enabled by recent anthropogenic climate change. This is especially demonstrated by growing regional disparities in soybean production across changing climate zones (Caetano et al., 2018). In relevant studies, for example, soybean plants exhibit marked vulnerability to heat stress, especially during the reproductive phases (Xia et al., 2026; Poudel et al., 2024), with a potential global yield decline of approximately 3.1% for every 1 °C increase in growing-season temperature (Zhao et al., 2017). Furthermore, moisture stress arising from irregular precipitation cycles severely constrained physiological development and final crop yields of soybeans in Iran (Araji et al., 2018). Thus, with rising food demand, the nature of increasing climate variability underscores the urgent need for earlier and more informed agricultural planning of soybean production to secure the food system.

Traditional yield forecasting methods employ on site field surveys as an essential input; however, these surveys are often subjective and expensive to carry out. Accurate crop yield forecasting systems have become an essential instrument for optimizing resource allocation and agricultural planning. Previous forecasting research has explored the relationship between climate variability and soybean production for crop yield forecasting, often employing advanced modeling techniques that integrate meteorological and remote sensing data. Machine learning, and subsequently deep learning, models have thus been increasingly explored as a scalable method for yield prediction. To enhance predictive power, studies have augmented meteorological inputs with satellite-derived spectral observations (e.g., Stepanov et al., 2020; Sun et al., 2019). These implementations of satellite imagery usually improve predictive performance, though often at the expense of agronomic interpretability.

Most existing machine learning-based models focusing on meteorological inputs, despite recent advantages, rely on datasets spanning only 10 to 35 years. For instance, Torsoni et al. (2023) utilized machine learning alongside meteorological records spanning 2002 to 2021, while Zhuang et al. (2023) and Chen et al. (2024) leveraged longer historical baselines of 34 and 35 years, respectively. However, this limited temporal scope has often failed to capture the subtle, long-term trends characteristic of anthropogenic climate change. This thereby underscores the necessity of a large-scale study to robustly quantify the impact of specific climate shifts on agricultural outcomes.

In this study, we synthesized a century-long dataset of historical weather records (1927–2025) and county-level soil characteristics for 2,327 U.S. counties, along with their soybean yields, sourced from the NOAA National Centers for Environmental Information (NCEI), the United States Geological Survey (USGS), and the USDA National Agricultural Statistics Service (NASS). This extensive temporal and spatial coverage enabled a robust investigation into the impacts of long-term climate change on soybean productivity. By integrating deep learning (DL) architectures with SHAP analysis, we developed and optimized an explainable predictive modeling framework for crop yield forecasting at the county-level. Beyond providing accurate in-season yield forecasts, this framework identifies critical climatic drivers contributing to soybean yield, offering a platform for assessing the long-term climatic potential of various U.S. regions for high-yield production.

## Materials and methods

### Study area

This study employed a wide temporal and spatial collection range for collecting environmental and yield data. We utilized a comprehensive dataset comprising weather, soil, and annual soybean yield records across the continental United States, spanning the period 1927–2025. This range, wider than those currently collected via remote sensing methods, allowed for the later developed machine learning framework to gain deeper insights into climate-yield relationships.

### Soybean yield attributes

Soybean yield data were acquired from the QuickStats Database,^1^ managed by the National Agricultural Statistics Service (NASS) of the United States Department of Agriculture (USDA). The dataset was constructed by querying “Soybeans” as the commodity, “Yield, Measured in Bu/Acre” as the data item, and “County” as the geographical level, encompassing all available states and years. This retrieval process yielded a total of 126,692 county-year observations. All yield metrics, originally recorded in bushels per acre (bu/acre), were retained for analysis, noting that one bushel per acre is approximately equivalent to 67.25 kilograms per hectare.

### Climate and weather attributes

Monthly climate and weather data were obtained from the Global Summary of the Month (GSOM) database (Lawrimore et al., 2016), maintained by the National Centers for Environmental Information (NCEI) of the National Oceanic and Atmospheric Administration (NOAA).^2^ Counties were georeferenced using latitude and longitude coordinates and assigned county names. Where multiple station observations were present within a single county, we computed a spatially averaged value. The dataset comprised 31 distinct climate variables processed on a monthly basis, categorized into 16 temperature-related, 8 precipitation-related, 7 snow- or freeze-related indicators. The details of these variables can be found in Table 1. By disaggregating these across the 12-month calendar year, a climate dataset totaling 372 monthly weather attributes was built to capture intra-annual climate dynamics.

**Table 1:** A total of 31 climate factors used in this study.

Climate factor	Description
CDSD	Cooling degree days to date
CLDD	Cooling degree days within month
DP01	Number of days with precipitation of greater than or equal to 0.254 millimeters
DP10	Number of days with precipitation of greater than or equal to 2.54 millimeters
DP1X	Number of days with precipitation of greater than or equal to 25.4 millimeters
DSND	Number of days with snow depth of greater than or equal to 25 millimeters
DSNW	Number of days with snowfall of greater than or equal to 25 millimeters
DT00	Number of days with maximum temperature of less than or equal to −17.8° C
DT32	Number of days with minimum temperature of less than or minimum to 0° C
DX32	Number of days with maximum temperature of less than or equal to 0° C
DX70	Number of days with maximum temperature of greater than or equal to 21.1° C
DX90	Number of days with maximum temperature of greater than or equal to 32.2° C
DYFG	Number of days where one or more instances of fog are reported
DYNT	Day of month with extreme minimum temperature
DYSD	Day of month with highest daily snow depth
DYSN	Day of month with highest daily snowfall
DYTS	Number of days where one or more thunderstorms are reported
DYXP	Day of month with highest daily total of precipitation
DYXT	Day of month with extreme maximum temperature
EMNT	Lowest daily minimum temperature of month
EMSD	Highest daily snow depth within month
EMSN	Highest daily snowfall within month
EMXP	Highest daily total of precipitation within month
EMXT	Extreme maximum temperature of month
HDSD	Heating degree days to date
HTDD	Heating degree days within month
PRCP	Total monthly precipitation
TAVG	Average monthly temperature
TMAX	Average monthly maximum temperature
TMIN	Average monthly minimum temperature
SNOW	Total monthly snowfall

Monthly data for each (JAN-DEC) was retrieved from the Global Summary of the Month (GSOM) database.

### Soil attributes

To complement the meteorological dataset, soil and topographic characteristics were collected and aggregated to the county level from SSURGO Averages Database,^3^ maintained by Natural Resources Conservation Center of the US Department of Agriculture of US Department of Agriculture (Wieczorek, 2014). For each county in our study area, we integrated a comprehensive suite of 15 soil variables (see Table 2) alongside one primary elevation metric. These data points were used to characterize the land-surface attributes, enabling the model to account for the interplay between topographical relief, soil-water holding capacity, and soybean yield potential. In addition, we calculated the average elevation using the measured elevations of each station within a county; this elevation was used as an additional input.

**Table 2:** A total of 15 soil attributes used in this study.

Soil factor	Description
AWC	Area and depth-weighted available water capacity
OM	Area and depth-weighted organic matter content in percent by weight
KFACT	Area and depth-weighted soil erodibility factor
BD	Area and depth-weighted bulk density in grams per cubic centimeter
KSAT	Area and depth-weighted saturated hydraulic conductivity
KV	Area and depth-weighted vertical saturated hydraulic conductivity
POR	Area and depth-weighted porosity
FC	Volumetric content of soil water retained at tension of 33 kPa
SILT	Area and depth-weighted average percent silt
SAND	Area and depth-weighted average percent sand
CLAY	Area and depth-weighted average percent clay
NO4	Area and depth-weighted fraction of soil passing through a No. 4 mesh sieve
NO10	Area and depth-weighted fraction of soil passing through a No. 10 mesh sieve
NO200	Area and depth-weighted fraction of soil passing through a No. 200 mesh sieve
THK	Horizon thickness

Retrieved from the USGS SSURGO database.

### Data and modeling preparation

The input features for developing predictive models comprised a total of 388 environmental factors, including 372 monthly climate factors, 15 soil attributes and one elevation variable, supplemented by a one year lagged county-level soybean yield to account for temporal technological and management differences. Initially, data covering the period 1927–2025 were compiled for 2,327 counties with weather data available. Data interpolation is often used to increase the number of data samples; however, with interpolation, we risk generating systematic bias that skews identified climate-yield relationships. Thus, to ensure model integrity, we instead employed listwise deletion for all records containing any missing predictor variables. Out of the 2,327 counties, 611 did not have any record of soybean yields, leaving 1,716 that have available yield data. Consequently, applying listwise deletion based on missing predictors resulted in a final dataset of 1,309 counties, representing 76.3% of the 1,716 counties with available yield data, and 23,748 county-year observations for analysis. Notably, more than 90% of the counties in the major soybean-producing states (such as Illinois, Indiana, and Iowa) were retained (see Supplementary Table 1), indicating that the analyzed dataset remained spatially representative of the major soybean-producing regions.

The data of 1927–2023 were then split randomly into training (90%) and validation (10%). The training set was utilized for model construction and weight optimization; the validation set served as a dedicated subset for hyperparameter tuning and architectural refinement. The data of 2024–2025 was reserved as an independent hold-out test set to provide an unbiased evaluation of the final model’s predictive accuracy and generalization performance.

### Feature preprocessing

Using a large number of insignificant variables in yield prediction could risk both overfitting to unrelated noise in training data and diluting the predictive signal of significant climate drivers. Thus, we employed a two-stage approach in our feature selection to target these issues. First, low variance factors were filtered out in order to reduce noisy variables. To reduce highly correlated features, correlational analysis was conducted via Pearson’s correlation coefficient. Features that were highly correlated with each other were removed based on the following criteria: first, the feature that appeared earliest (May vs. July) would be kept, then features with demonstrated biological impact to soybeans in literature would be kept, and finally whichever feature had a higher individual correlation with yield through a single linear regression was kept. Thus, we were able to filter the total amount of variables studied and reduce variable collinearity.

### SHAP analysis

To evaluate the contribution of individual environmental factors to the soybean yield predictive model, we first utilized SHapley Additive exPlanations (SHAP), a method from cooperative game theory that measures the average magnitude of each feature’s effect on model predictions (Lundberg and Lee, 2017). SHAP ensures model interpretability by fairly distributing the model’s predicted yield among all input features (Mosca et al., 2022). Unlike traditional importance metrics, SHAP provides both local and global transparency by aggregating these local SHAP values across the entire training set. This approach enables identifying the specific climatic and soil factors—such as temperature indices, soil moisture levels, and elevation—that most significantly influence soybean yield variability, while also clarifying the directional impact of these variables on model outcomes.

Through this study, we utilized the Shapley analysis to identify contributing factors to soybean yield. Because exact TreeSHAP values are available for tree-based models, we trained a Random Forest using the same predictor variables, response variable, preprocessing pipeline, and training data as the DNN. The Random Forest served as an interpretable companion model for identifying dominant predictor-response relationships. The SHAP analysis was not intended to explain the internal computations of the DNN directly but rather to determine whether an independent nonlinear model identified the environmental drivers of soybean yield. All of the significant environmental variables identified through the TreeSHAP analysis were utilized as inputs within the modeling and training for the deep neural network.

### Deep neural network for modeling soybean yield prediction

Deep neural networks (DNNs) represent a class of machine learning models that utilize layered architectures of interconnected neurons to perform complex predictive tasks (Sarker, 2021). Recent research has demonstrated that DNNs frequently outperform traditional machine learning methodologies in agricultural yield forecasting (Sun et al., 2019; Lu et al., 2024). We leveraged these models primarily due to their ability to model non-linear, high-dimensional relationships between climate indicators and crop yields.

After filtering factors, we utilized a fully connected deep neural network for yield prediction. Here, to optimize key hyperparameters, we performed a grip searching including learning rate, including learning rate, activation functions, and neuron configuration. These parameters were tuned based on performance metrics of Mean Square Error (MSE) derived from the validation dataset. To ensure model robustness and mitigate overfitting, we utilized the ADAM optimizer and calibrated the number of training epochs to achieve peak generalization performance. Following training, the model was evaluated using an independent hold-out test set, with predictive performance quantified via the coefficient of determination (R^2^).

### Evaluation of model performance

We used R^2^ within this study for assessing the prediction performance of machine learning and deep learning models constructed throughout this study. The metric is able to measure how well the model is able to explain the variance between its predicted yields and the actual observed yield for a given county-year instance. The mathematical formula is shown in the following equation:

$$R^{2}=1-\frac{\sum_{i=1}^{n}{\left(y_{i}-\hat{y_{i}}\right)}^{2}}{\sum_{i=1}^{n}{\left(y_{i}-\underline{y}\right)}^{2}}$$

Where $\hat{y_{i}}$ is the model predicted yield, $y_{i}$ is the measured yield, $\underline{y}$ is the average yield, *n* is the total number of samples.

## Results

### Exploratory analysis of climate data

We began by analyzing the underlying trends within the data which could shape potential climate-yield interaction. Exploratory analysis of the 372 monthly weather attributes revealed two distinct seasonal trajectories. Heat stress and precipitation-related variables exhibited a consistent bell-shaped distribution, with peak values occurring during the middle months, reflecting the annual thermal cycle and heightened evaporation. In contrast, freeze-related variables exhibited an inverse U-shaped distribution, reaching their maxima during the winter season (Figure 1).

**Figure 1:**
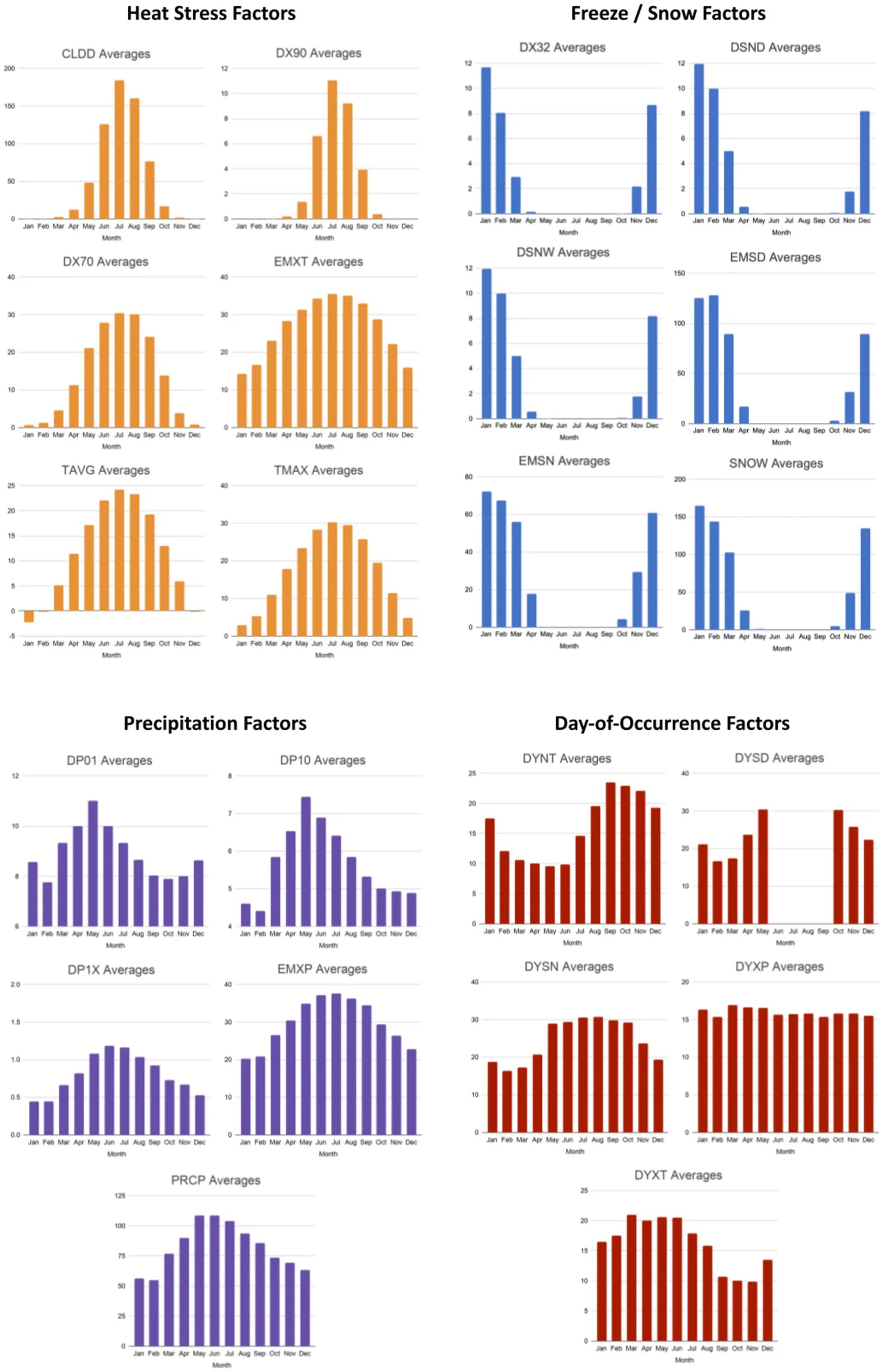
Selected climate factors distribution across months. These graphs show the distribution of value averages across different months for selected climate features, including temperature, precipitation, and freeze factors. For months in which a given variable was not applicable or no events were observed, the corresponding values were coded as 0.

### SHAP analysis of contributing factors for soybean yield

We utilized SHAP analysis to evaluate the contribution of the individual features to the soybean yield. By substituting the final model for a random forest regressor, which matches the final model in representing non-linear relationships, we enable rapid and accurate measurements of feature contributions associated with soybean yield. Figure 2 presents a beeswarm plot illustrating the distribution of SHAP values for the top 20 ranking variables by mean absolute SHAP value. Notably, our analysis reveals a high concentration of predictive power in late summer climate attributes: five of the top ten contributors originate from August (DX90, TMAX, CLDD, EMXT, and EMNT), followed by two from July (TMAX, DX90), one each from September (EMNT) and one from October (DX70), and one soil-based variable (AWC). In addition, the top four features all represented August heat stress factors. This temporal clustering underscores the sensitivity of soybean reproductive phases, particularly grain filling and maturation, to late summer climatic conditions.

**Figure 2:**
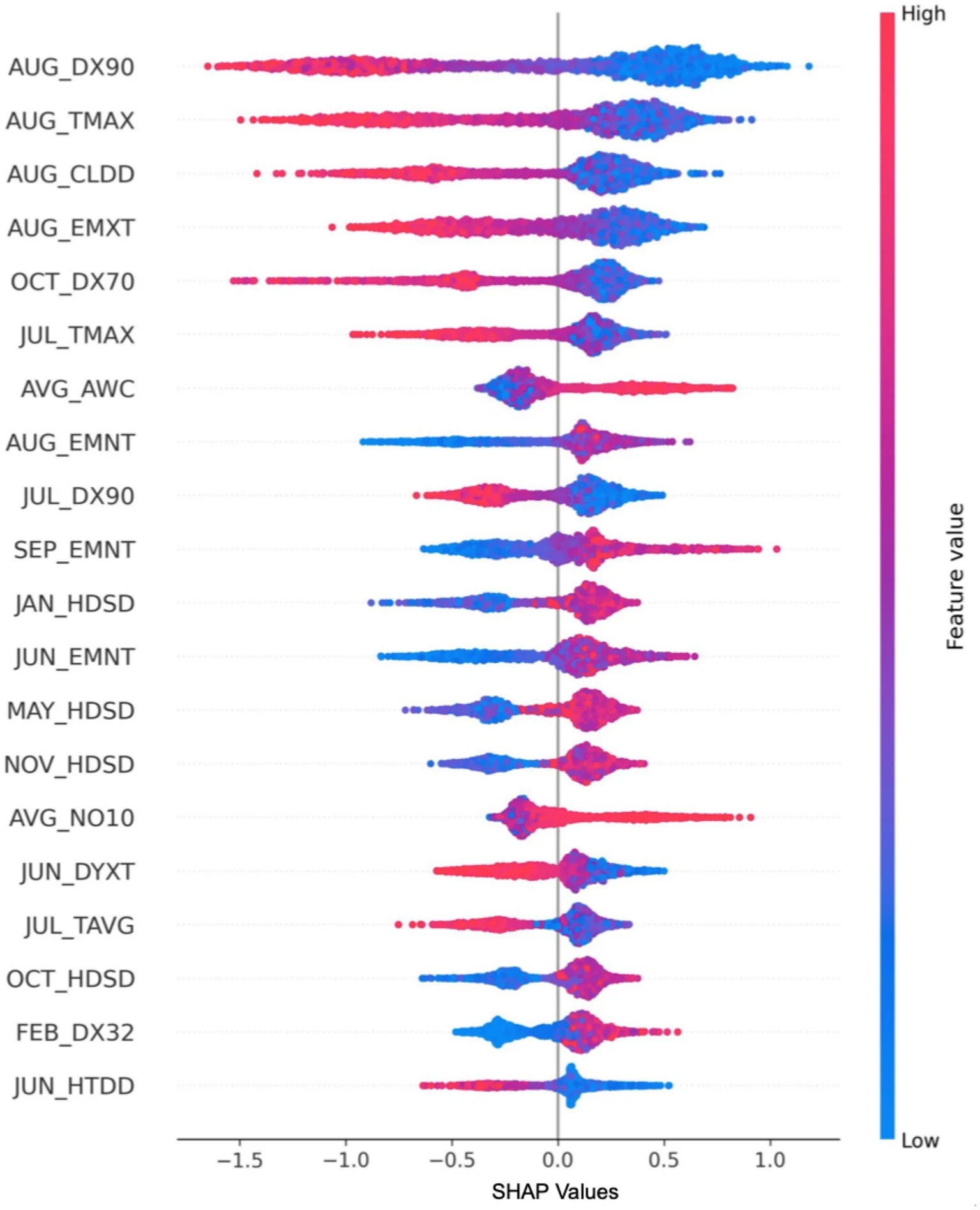
Beeswarm plot of local SHAP values for top twenty independent variables. The x-axis represents the SHAP value for a given prediction. A positive and higher SHAP value indicates the given factor contributed positively and in a large amount towards yield. Each dot within that row represents the SHAP value for that variable on a given local prediction. The color of each dot denotes the value of the variable for the given SHAP value. Soil attributes all start with “AVG_”.

Further analysis of the SHAP values for weather variables on their marginal contribution to yields showed several different patterns (Figure 3). The first cluster, which comprised of factors with negative marginal contributions during the critical mid-to-late growing season (June-September), included the number of days with maximum temperatures 32.2 °C (DX90), and average annual maximum temperature (TMAX), cooling degree days (CLDD), and extreme monthly maximum temperature (EMXT), and average monthly temperature (TAVG). These variables noticeably demonstrated peaks in July or August, indicating that heat stress during the reproductive and grain-filling stages exerts a substantial negative impact on final soybean yields. Conversely, the second cluster, consisting of total monthly precipitation-related variables (PRCP, DP01, DP10, and EMXP), lowest daily minimum temperature (EMNT), and heating degree days (HDSD), displayed a coherent positive pattern between June and October. This suggests that adequate moisture availability and moderate temperature ranges during these months are essential for maximizing physiological development and final output. Other clusters included the variables with mixed or minimal marginal effects in June to September and those with negative effects in March to May.

**Figure 3:**
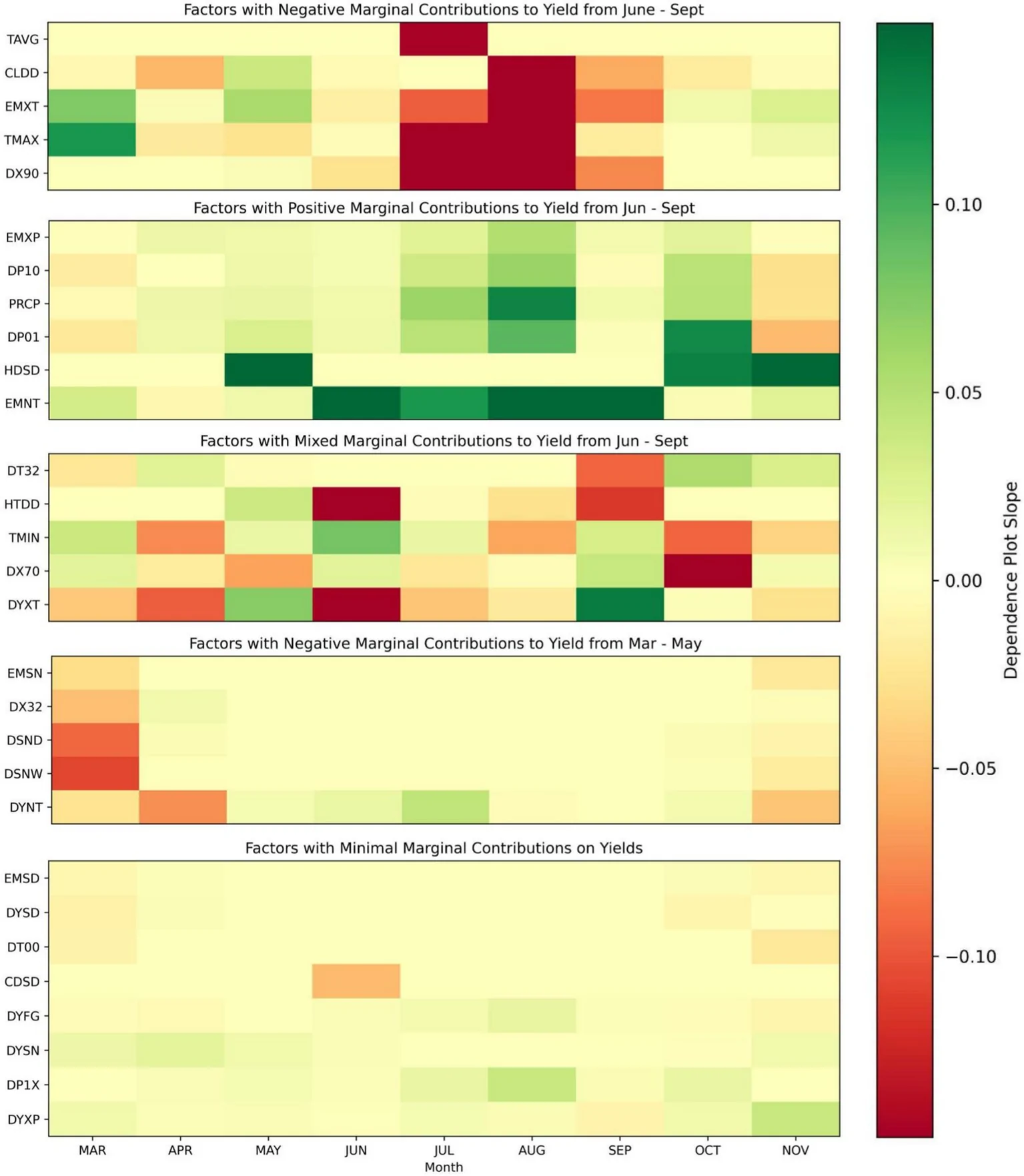
Heatmap of averaged marginal contribution on yield for climate variables across growing months (March – November) based on SHAP analysis. This heatmap shows how each variable may contribute towards yield predictions. The y-axis represents various climate factors, while the x-axis represents month of occurrence. The color of each box represents the slope values derived from the dependent plot of SHAP values for each variable on its contribution to yield prediction; red indicates a negative relationship with yield, where a higher value will contribute towards less yield; green represents a positive relationship with yield.

Partial dependence analysis further provided nuanced insights into the relationship between specific climatic variables and soybean yields. Several distinct threshold patterns are shown in Figure 4. For instance, it was shown DX90 in August exerted a significant negative influence on yield; while values below 7 days correlate with positive yield outcomes, which values over 12 days trigger a decline, with the magnitude of this negative impact intensifying as the number of days rose. Conversely, other variables exhibit linear or threshold-based positive correlations. Total monthly precipitation (PRCP) shows a clear positive dependency; values below 90 mm generally constrain yields, whereas PRCP levels above 100 mm demonstrate a consistent positive effect. Furthermore, some variables exhibit a bell-shaped impact on productivity, indicating an optimal range. TMAX in September exemplifies this pattern: yield is positively correlated with temperatures ranging between 22.5 °C and 28.5 °C, but shifts to a negative impact when temperatures fall outside this optimal physiological window.

**Figure 4:**
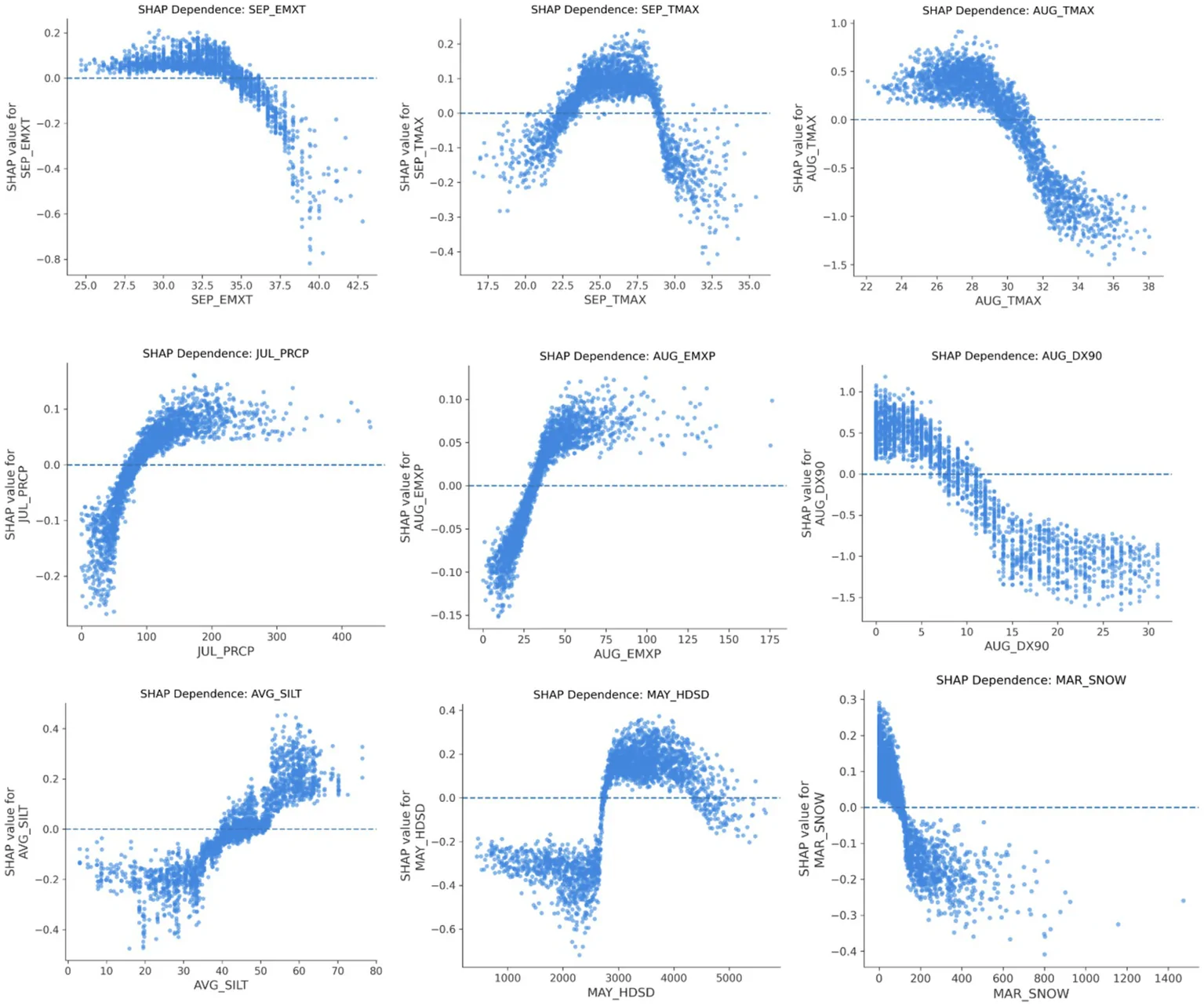
SHAP dependence plots for selected significant climate variables based on marginal contribution to yield prediction. These graphs show the relationship between the values of nine factors with significant marginal contributions towards yield predictions and their local contribution towards yields shown through SHAP, with each value-SHAP pair represented as an individual point. The x-axis represents the value of the variable for each data point, while the y-axis represents the corresponding SHAP values. In general, a positive slope represents a positive relationship between the variable and yield, where a higher value for the given variable leads to an increase in the predicted yield for the model. Variables are named using the abbreviation of the month in which they were collected, followed by the variable name by the final four characters.

### Deep neural network model for soybean yield forecasts

The developed DNN model was initially assessed using the validation set, and the main parameters was shown in Supplementary Table 2. When applying the model incorporating the monthly meteorological and soil attributes, we observed a strong alignment between predicted and actual soybean end-of-year yields, suggesting that the DNN framework effectively captures the variance in county-level soybean productivity and provides robust predictive accuracy.

Furthermore, the DNN model was evaluated using the hold-out test dataset for 2024 and 2025 retrieved from USDA-NASS reports. When applying the model incorporating the full suite of monthly meteorological and soil attributes, we observed a strong alignment between predicted and actual soybean end-of-year yields. The model achieved a coefficient of determination R^2^ of 0.668 and 0.678 in 2024 and 2025, respectively, showing consistent predictions in hold-out testing (Figure 5; Supplementary Table 3). A sensitivity analysis was conducted to exclude one-year lagged soybean yield from the DNN model, and it resulted the coefficient of determination of R^2^ of 0.3154 and 0.3515 in 2024 and 2025. These results suggest both the temporal trends and environmental data contribute to model prediction for soybean yield.

**Figure 5:**
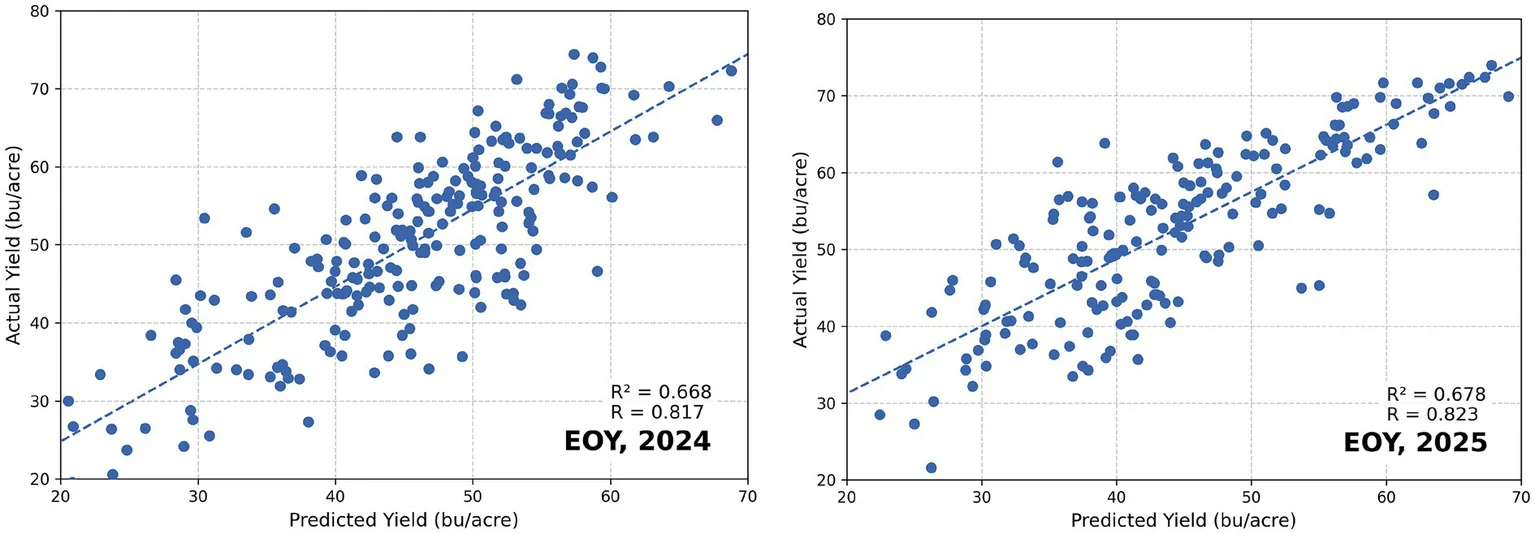
Actual vs. predicted yield plot for deep neural network soybean predictions of end-of-year (EOY) yields on hold-out test set of the years 2024 and 2025. In this graph, x-axis represents predicted yields, and y-axis represents corresponding actual yields. End-of-year prediction use all monthly factors and is normally standard for post-harvest analysis of crop yields. Each point shown represents a single county-year instance.

We further examined the spatial residuals, which are defined as the differences between predicted and actual yields, to determine the predicted error geographically. As shown in Supplementary Figure 1, the main soybean producing states, including Iowa and Illinois exhibited relatively small residuals, indicating good agreement between predicted and actual yields. By contrast, the Mid-Atlantic region, including the state of Pennsylvania, tended to be overestimated, while states such as Wisconsin and Tennessee showed a greater tendency toward underestimated yields.

To evaluate the model’s capacity for early forecasting, we performed in-season predictions using cumulative monthly weather data in 2025. Figures 6A–D illustrate model performance when integrating data from May through August. As expected, models using cumulative data through May, June, July and August showed increasing predictive power, with R^2^ values of 0.516, 0.596, 0.622, and 0.668, respectively—only slightly lower than the full-year model (R^2^ = 0.678). Similar observations were found in 2024. Notably, when incorporating July or August weather data, the R^2^ reached close to the full-year performance. This result aligns with our SHAP analysis, which identified these two months as the critical window for soybean grain filling and maturation. These findings confirm that our DNN model is capable of providing reliable, in-season soybean yield forecasts as early as May, offering a robust tool for agricultural stakeholders to adjust management strategies well before harvest.

**Figure 6:**
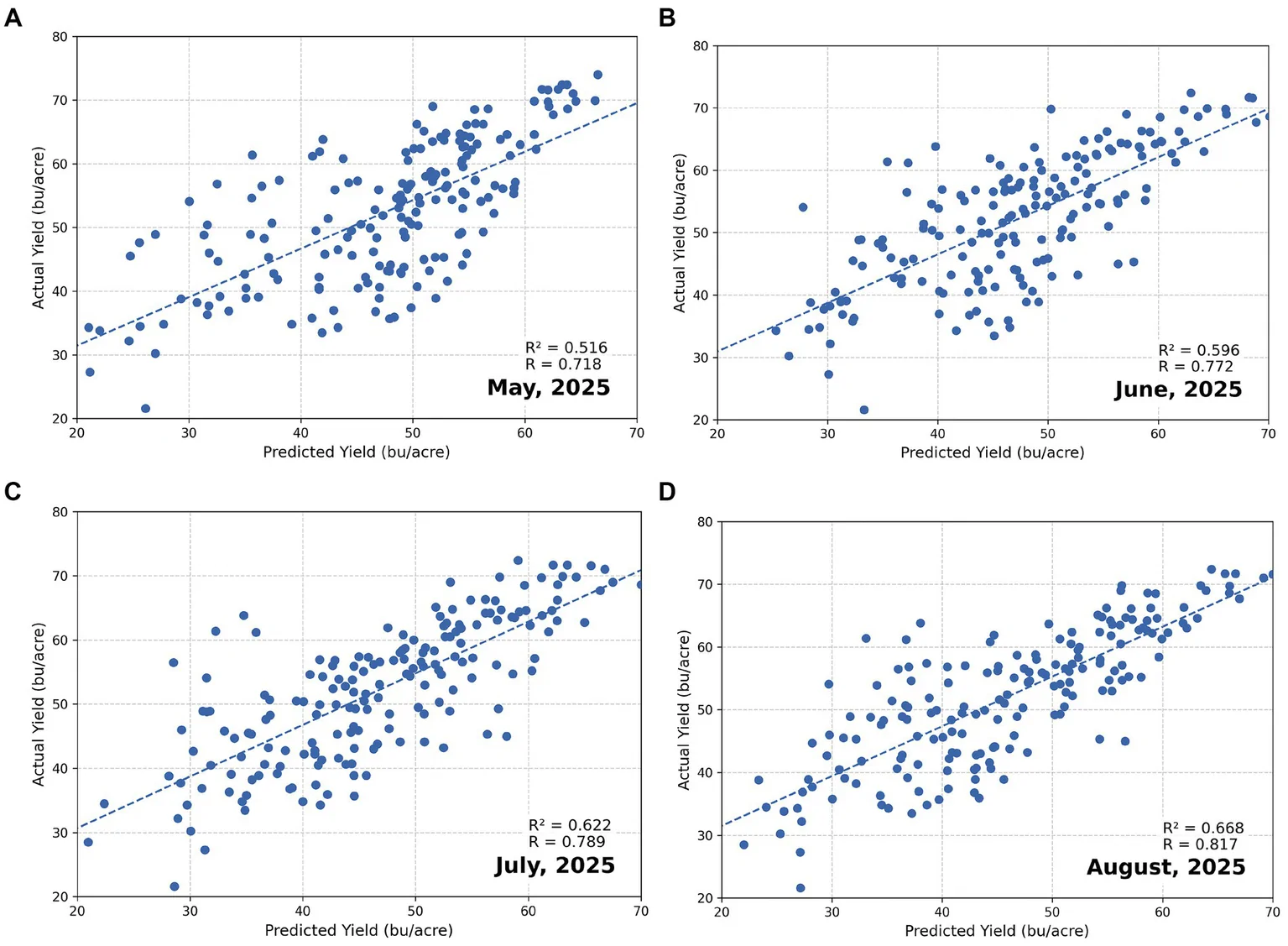
Actual vs. predicted yield plots for in-season deep neural network soybean forecasts on hold-out test set of 2025. These graphs show the predictive accuracy for different monthly yield forecasting models. The x-axis represents predicted yields, while the y-axis represents corresponding actual yields. Top left **(A)** May, using monthly factors collected in May or before (R^2^ = 0.516); top right **(B)** is June (R^2^ = 0.596); bottom left **(C)** is July (R^2^ = 0.622); bottom right **(D)** is August (R^2^ = 0.668).

## Discussion

In this study, we synthesized county-level weather, soil, and yield data spanning 1927 to 2025 to evaluate the impact of various monthly weather indicators on soybean yields forecasts using a combination of statistical and deep learning methodologies. Through this, we trained a deep learning neural network driven by environmental factors to forecast country-level soybean yields, archiving an R^2^ value of 0.668 and 0.678 against observed yields in the hold-out test set of the year 2024 and 2025, respectively. SHAP analysis revealed that optimal soybean growth is associated with fewer extreme heat days (DX90, EMXT), lower daily maximum temperatures (TMAX), and soils with high water-holding capacity (AWC).

Several comparable models have been developed to predict regional soybean yields in literature; these typically rely on limited temporal or spatial scales and data-heavy remote sensing data. For example, Lu et al. (2024) developed a GCBA predictive model using multi-source remote sensing and monthly climate data from 1,085 counties over the period 2008–2020, achieving an R^2^ of 0.68 and 0.70 when tested on the hold-out data in 2019 and 2020, respectively. In contrast, our study leveraged an extensive dataset from primarily monthly climate and soil data, encompassing 1,309 counties and nearly a century of data (1927–2025), to train deep learning neural network for yield forecasting. Our model achieved a comparable performance at a R^2^ of 0.668 and 0.678 in 2024 and 2025, respectively. This climate-based framework maintains accuracy while sacrificing spatial resolution for three distinct advantages. First, the extensive range of data, enabled by long meteorological record availability, allows the model to learn from a much broader range of climate variability that may be underrepresented in a shorter data record. Second, because our approach relies on climate data rather than satellite imagery, it enables yield estimates as early as May, significantly ahead of remote sensing pipelines. And third, this approach preserves agronomics interpretability, as each input variable corresponds to a physically meaningful climate driver. These characteristics suggest that climate-based approaches may be able to fill gaps found in remote sensing-based yield forecasting pipelines, allowing for early and comprehensive estimations.

Our model demonstrated utility for in-season yield forecasting by providing reliable estimates well before the harvest period. Our framework achieved robust predictive performance with an R^2^ of 0.516 as early as May, increasing to 0.596 in June, 0.622 in July, 0.668 in August, and reaching a peak R^2^ of 0.678 for end-of-year forecasts. This performance is comparable to, and even outperforms, other reports in literature; for instance, Khaki et al. (2021) reported an R^2^ of 0.50 (R of 0.71) in July and R^2^ of 0.59 (R of 0.77) in August in their hold-out test. By maintaining high accuracy with greater lead times, our model offers a superior tool for early-season agricultural decision-making and risk management in soybean production.

The SHAP analysis identified that the top five predictors of soybean yield are primarily related to summer temperature dynamics; including number of days exceeding 32 °C (DX90), average monthly maximum temperatures (TMAX), and monthly extreme maximum temperatures (EMXT). These findings suggest that the climatic shifts associated with global warming are shifting more favorable thermal environments for soybean cultivation toward higher latitudes.

Our research further identifies the temporal occurrence of the monthly maximum temperature, which is defined as the day within a given month when the peak temperature occurs (DYXT), as a potential indicator of yield variation. Our findings suggest that a higher DYXT value in June (i.e., a later occurrence in month of the hottest day) is negatively correlated with soybean yields, whereas a higher DYXT value in September is positively associated with increased yields. These results suggest that the duration and timing of the growing season are another influential factor as absolute thermal intensity; specifically, an extended, well-timed summer period appears to be more conducive to higher aggregate yields than one characterized by early-season heat stress.

Furthermore, our SHAP analysis also revealed a bell-shaped relationship for September maximum temperature (TMAX) (Figure 4) with an optimal physiological window of approximately 22.5–28.5 °C, which is consistent with soybean temperature requirements during the grain-filling stage. During this critical period, moderate daytime temperatures could maximize photosynthetic carbon assimilation while maintaining Photosystem II function and chloroplast integrity and promoting efficient seed development (Ding et al., 2024). In contrast, higher temperatures above the optimal range could reduce photosynthetic efficiency and carbon fixation through heat stress, limiting assimilate availability for grain filling (Ding et al., 2024). Elevated temperatures also increase respiratory carbon loss, depleting carbohydrate reserves needed for seed filling (Yang et al., 2023). These physiological mechanisms explain the bell-shaped response identified in our model, while soybean yield declines outside the optimal September temperature range.

Moisture availability has long been established as a critical determinant of soybean productivity (Hill et al., 1979). In our study, we found that soil available water storage capability (AWC) emerged as the sixth most significant predictor of yield, following five temperature-related variables. We hypothesize that this prioritization of temperature over moisture in our model arises from two primary factors. First, the most productive soybean regions (such as Illinois, Ohio, and Michigan) are situated near the Great Lakes, a region characterized by consistent moisture availability; the other climate-potential regions, such as New York and Pennsylvania, share similar proximity to these moisture-rich basins. Second, the widespread adoption of advanced irrigation systems has largely mitigated water-limiting constraints in modern agriculture. Conversely, temperature remains an exogenous, uncontrollable variable; thus, it exerts a more decisive influence on yield variability than soil moisture in modern production environments.

While this study provides significant insights into climate-driven soybean yield variability, several limitations warrant consideration. First, as a retrospective analysis, our model requires prospective validation when confirming its predictive utility across future climate scenarios. Second, the reliance on county-level data, while robust for regional assessment, lacks the granularity of field-level observations; future research should aim to incorporate precision agricultural data to bridge this gap. Third, although our model integrates weather and soil variables, soybean yield is influenced by a complex nexus of factors, including fertilizer application, agronomic management practices, and cultivar selection, which are not fully represented individually due to data availability restrictions. Fourth, some counties had missing values in environmental or yield data and were not included in the analysis, which reduced the coverage of our dataset and model performance. Finally, the scope of our study is limited to the continental United States. Future work should expand the model to include other major soybean-producing nations, such as Brazil, to test the global applicability of these temperature-sensitive indicators and temporal metrics.

## Conclusion

In summary, this study introduces a century-scale deep learning framework capable of robust, in-season soybean yield forecasting at the county level across the United States using climate data. These findings offer significant practical utility for stakeholders; by providing early-season yield projections, the model enables farmers to optimize management strategies and assists policymakers in enhancing food distribution and supply chain resilience. Moreover, the underlying deep learning architecture is inherently transferable, offering a scalable template that can be adapted to evaluate climate-change impacts on other staple crops or extended to diverse global agricultural landscapes. Ultimately, this research provides a data-driven approach to bolster food security and mitigate the risks associated with climate-induced volatility in modern agriculture.

## Data Availability

The original contributions presented in the study are included in the article/Supplementary material, further inquiries can be directed to the corresponding author.

## Footnotes

1
https://quickstats.nass.usda.gov/

2
https://www.ncei.noaa.gov/access/metadata/landing-page/bin/iso?id=gov.noaa.ncdc:C00947

3
https://www.nrcs.usda.gov/resources/data-and-reports/soil-survey-geographic-database-ssurgo
