# Supplementary material for: A deep learning approach to county-level soybean yield forecasting using large-scale environmental data
Source: Front Artif Intell. 2026 Sep 17;9:1914697. doi: 10.3389/frai.2026.1914697 (PMC13627415; doi:10.3389/frai.2026.1914697)
Supplement: Supplementary file 1 [file Data_Sheet_1.docx]

Supplemental Table 1. The counties of the US states before and after listwise deletion in the analysis.

| States | Original Counties | Counties after Listwise Deletion | Percentage |
| --- | --- | --- | --- |
| ALABAMA | 64 | 47 | 73% |
| ARKANSAS | 70 | 51 | 73% |
| DELAWARE | 2 | 2 | 100% |
| FLORIDA | 27 | 11 | 41% |
| GEORGIA | 112 | 49 | 44% |
| ILLINOIS | 87 | 81 | 93% |
| INDIANA | 83 | 81 | 98% |
| IOWA | 92 | 91 | 99% |
| KANSAS | 105 | 98 | 93% |
| KENTUCKY | 75 | 37 | 49% |
| LOUISIANA | 41 | 29 | 71% |
| MARYLAND | 19 | 19 | 100% |
| MICHIGAN | 67 | 51 | 76% |
| MINNESOTA | 74 | 65 | 88% |
| MISSISSIPPI | 74 | 52 | 70% |
| MISSOURI | 102 | 84 | 82% |
| NEBRASKA | 84 | 75 | 89% |
| OHIO | 83 | 70 | 84% |
| OKLAHOMA | 75 | 50 | 67% |
| PENNSYLVANIA | 55 | 53 | 96% |
| TENNESSEE | 76 | 55 | 72% |
| TEXAS | 112 | 43 | 38% |
| VIRGINIA | 72 | 52 | 72% |
| WISCONSIN | 65 | 63 | 97% |
|  |  |  |  |
| Summary | 1716 | 1309 | 76.3% |

Supplemental Table 2. The final optimized hypermeters of the DNN model

Input layer: 116

Output layer: 1

Number of layers: 8

Neurons per layer: 512,512,512, 512, 256,256,256,256

Learning rate: 0.001

Activation functions: Rectified Linear Unit (ReLU)

Supplemental Figure 1. The geographical residuals of actual yields and prediction from the DNN model. The red color means predicted yield is higher than the actual yield, while the green color represents visa versa.
